# Early Heat Exposure Effects on Proteomic Changes of the Broiler Liver under Acute Heat Stress

**DOI:** 10.3390/ani11051338

**Published:** 2021-05-08

**Authors:** Darae Kang, Kwanseob Shim

**Affiliations:** 1 Department of Animal Biotechnology, College of Agriculture Life Science, Jeonbuk National University, Jeonbuk 54896, Korea; kangdr92@gmail.com; 2 Department of Agricultural Coveragence Technology, College of Agriculture Life Science, Jeonbuk National University, Jeonbuk 54896, Korea

**Keywords:** acute heat stress, broiler, early heat exposure, liver, proteomics

## Abstract

**Simple Summary:**

Early heat exposure have been studied in the poultry industry as a method of reducing heat stress (HS) on poultry. However, the results of each study are inconsistent, and it has not been confirmed which mechanisms reduce HS by early heat exposure. Therefore, we tried to confirm the relaxation mechanism through proteomic analysis after applying early and acute heat exposure to broilers. The broilers were divided into three treatments, followed by CC (control group), CH (acute HS at the 35th day), and HH (early heat exposure at the fifth day and acute HS at the 35th day. Liver samples were collected and analyzed for proteomics and functional analysis. Proteins related to various functions, such as carbohydrate metabolism, fatty acid metabolism, energy metabolism, and the oxidation–reduction process, which were dramatically changed by acute HS, and were alleviated similar to the control group by early heat exposure. Through these results, the mechanism by which early heat exposure induces homeostasis during acute HS, and the possibility of the early heat exposure as a method of reducing HS were confirmed.

**Abstract:**

As environmental temperatures continue to rise, heat stress (HS) is having a negative effect on the livestock industry. In order to solve this problem, many studies have been conducted to reduce HS. Among them, early heat exposure has been suggested as a method for reducing HS in poultry. In this study, we analyzed proteomics and tried to identify the metabolic mechanisms of early heat exposure on acute HS. A total of 48 chicks were separated into three groups: CC (control groups raised at optimum temperature), CH (raised with CC but exposed acute HS at the 35th day), and HH (raised with CC but exposed early heat at the fifth day and acute HS at the 35th day). After the whole period, liver samples were collected for proteomic analysis. A total of 97 differentially expressed proteins were identified by acute HS. Of these, 62 proteins recovered their expression levels by early heat exposure. We used these 62 proteins to determine the protective effects of early heat exposure. Of the various protein-related terms, we focused on the oxidative phosphorylation, fatty acid metabolism, carbohydrate metabolism, and energy production metabolism. Our findings suggest the possibility of early heat exposure effects in acute HS that may be useful in breeding or management techniques for producing broilers with high heat resistance.

## 1. Introduction

Over time, global warming has become one of the major concerns facing livestock in terms of their welfare and productivity. The comfortable temperature for poultry is approximately 22 °C, and heat stress (HS) occurs at approximately 32 °C [1]. Unlike other animal species, poultry are susceptible to HS because they are covered with feathers, have no sweat glands, and have a high basal body temperature of 41 °C. These characteristics make heat dissipation difficult [2]. As an immediate response to excessive heat in broilers, body temperature rises rapidly, and hyperventilation (panting) occurs to dissipate or maintain thermal homeostasis [3]. High body temperature reduces the release of thyroid hormones, such as triiodothyronine (T3) and thyroxine (T4), which promote chicken growth [4]. In addition, acute HS affects metabolism and induces oxidative protein and lipid damage [5,6]. Therefore, in an effort to reduce HS in poultry, various studies on management, poultry facilities, feed or water additives, breeding, and early heat exposure have been conducted [1,7,8]. Among those various methods, the early heat exposure method is being applied as an efficient method that can easily reduce heat stress without controlling facilities or manipulating feed and water. Early heat during the incubation period or at 5 days old in the post-hatched period is applied to develop heat resistance before marketing. The main analysis factors in these studies were growth performance, physiological characteristics, hormones, specific proteins, and gene levels.

Proteomic and genomic analyses are used to analyze the response of metabolism to environmental stimulation, because they are rapid methods for whole-expression analysis. Proteins are generated based on genomic information, but expression level or protein types may vary depending on the process of transformation and metabolic mechanism after protein translation. In addition, proteins are important materials that act directly in the body. Following treatment, the differentially expressed proteins could be identified as specific proteins by stimulation and used as HS markers. To date, proteomes have been used to analyze differentially expressed proteins to investigate HS effects in several animals [6,9,10,11,12,13], and specific proteins responding to HS have been identified. These proteins are involved in ROS production, mitochondrial oxidation–reduction pathways, energy metabolism, and cell apoptosis [9,10,11,12,13], eliciting animal responses to external stress for protecting cells in the body and resist HS. However, studies to understand protein changes in broilers due to early heat exposure have seldom been conducted.

In this study, we hypothesized that the early heat exposure method is effective in relieving acute HS. Therefore, we performed proteomic analyses to identify the changes it causes. Broilers were exposed to early or acute HS, then the liver tissues were collected and subjected to proteomic analyses. Physiological and metabolic pathways were assessed using differentially expressed proteins (DEPs) to determine which pathways were affected by acute HS and ameliorated by early heat exposure.

## 2. Materials and Methods

The animal experimental procedures were approved by the Animal Ethics Committee of Jeonbuk National University (CBNU2018-097), the Republic of Korea.

### 2.1. Animals and Heat Exposure Conditions

**Figure 1:**
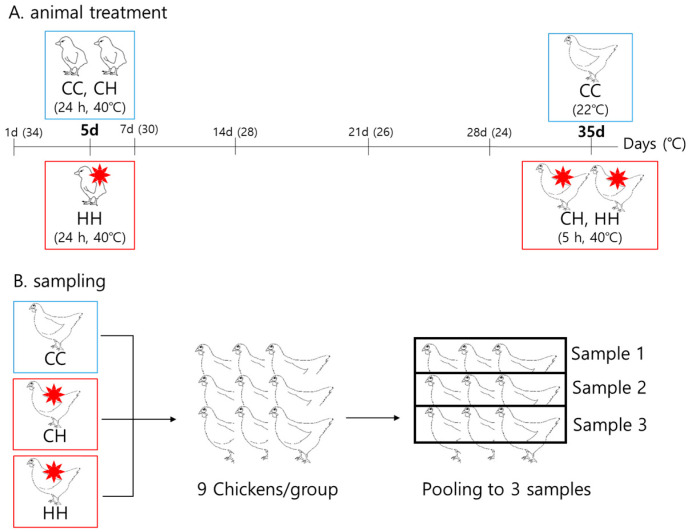
Heat exposure and sampling design for broilers. (**A**) Animal treatment design. (**B**) Sampling design. CC: raised at a convenient temperature without any heat exposure. CH: acutely heat-stressed broilers. HH: early heat exposure and acutely heat-stressed broilers. For the rest of the period not shown in the figure, all groups were kept in the control environment.

A total of 144 one-day-old Ross chicks were purchased from Dongwoo Hatchery (Iksan, Korea). The chicks were weighed and randomly grouped into three groups of chicks without significant weight differences. Each group had four replicates, with 12 chicks per pen. All chicks were raised at 34 °C from one day old, reduced by 2 °C weekly to 22 °C. Humidity was maintained at 57% ± 3%. All chicks had free access to water and feed (Table S1). The heat treatment conditions were as follows: the CC group, raised at a suitable temperature as explained above, without heat exposure; the CH group, raised at the same temperature as the CC group during the whole period except for acute heat exposure at 40 °C for 5 h at 35 days of age; and the HH group, raised at the same temperature as the CC group during the period, except for early heat exposure at 40 °C for 24 h at 5 days of age and acute heat exposure at 40 °C for 5 h at 35 days of age (Figure 1). At the end of the experimental period, the 27 chickens were weighed and sacrificed, and the liver tissues were collected, immediately frozen in liquid nitrogen, and stored at −80 °C until analysis. The growth performance of the chickens was analyzed in a previous study [14]. Nine samples per group were pooled by three to make the final three samples. Therefore, we used three pooled samples per each group for proteomic analysis.

### 2.2. Protein Extraction and Digestion

The same amount (100 mg) of chicken liver tissue for each broiler was lysed in 1 mL of 8 M urea and protein inhibitor by vortex mixing for 30 s and sonicating for 3 min in an ice bath. The tissue was homogenized, centrifuged at 14,000 rpm for 10 min, and the supernatant was collected. Protein concentration was measured using the BCA assay. Proteins from each tissue were used for in-solution digestion. Depleted protein (100 µg) in 100 mM Tris buffer (pH 8.0, total 30 µL) was incubated with 6 M urea and 20 mM DTT at 56 °C for 30 min and alkylated for 30 min with fresh 50 mM iodoacetamide in 100 mM Tris (pH 8.0) in the dark at room temperature. The reaction was quenched with 100 mM Tris (pH 8.0), and the protein was enzymatically digested using a trypsin/Lys-C Mix (1:50 enzyme/substrate) and incubated at 37 °C overnight. The reaction was quenched with formic acid, and the peptides were desalted using an HLB oasis column (Waters Corporation, Millford, MA, USA). The resulting peptides were dried using a speed vac.

### 2.3. LC-MS/MS Analysis

The EASY-nLC 1000 system (Thermo Fisher Scientific, Rockford, IL, USA) was used for peptide separation in a C18 column (2 µm particle size, 50 µm id × 15 cm length; Thermo Fisher Scientific) at a flow rate of 300 nL/min with mobile phases A (0.1% formic acid in water) and B (0.1% formic acid in 100% acetonitrile). The gradient profile was set as follows: 5–40% B for 45 min, and 40–80% B for 2 min. MS analysis was performed using a Q Exactive mass spectrometer (Thermo Fisher Scientific, Bremen, Germany) with the spray voltage set at 2.3 kV. MS spectra were collected at a resolution of 70,000 at 200 and 350–2000 *m*/*z* mass range, followed by data-dependent HCD MS/MS (at a resolution of 17,500 and collision energy 25%) of the 20 most abundant ions. A dynamic exclusion time of 30 s was used in this study.

Raw files for chicken liver protein identification were analyzed using Proteome Discoverer (version 1.4, Thermo Fisher Scientific) with a percolator against the UniProt Chicken database (https://www.uniprot.org/proteomes/UP000000539, accessed on 1 February 2021). Oxidation was chosen as a dynamic modification, and carbamidomethyl was chosen as a static modification. The mass error for the parent ion mass was ±10 ppm, with a fragment ion of ±0.02 Da. Peptides were searched for full tryptic specificity, allowing two missed cleavages. The search parameters used were a precursor tolerance of 10 ppm and 0.02 Da fragment ion tolerance. For label-free quantitation, Scaffold Q + S was used. All identified proteins were normalized by protein size and total spectrum count number, and normalized data were filtered with a confidence level (CI > 95%). For further filtering, the false discovery rate (FDR) was set to 0.05, and count number > 0 for two out of two replicates.

### 2.4. Gene Ontology Enrichment Analysis

Differentially expressed proteins were clustered using their coding genes. The bioinformatics programs used were DAVID Bioinformatics Resources 6.8 (https://david.ncifcrf.gov/, accessed on 1 February 2021) with the Gene Ontology database and ClueGo (Cytoscape 3.8.2) with the KEGG pathway database. These classifications were applied to the Gallus database.

### 2.5. Gene Expression by qPCR

Gene expression was analyzed to confirm the differentially expressed protein expression. RNA was isolated using Accuzol Total RNA extraction kit (Bioneer, Daejeon, Korea), and the concentration and purity were determined using a µDrop plate (NanoDrop; Thermo Fisher Scientific). From 1 µg of the total RNA, complementary DNA was synthesized using a cDNA synthesis kit (AccuPower Cycle RT premix, dt20; Bioneer, Daejeon, Korea). The primers were designed using Primer 3 software (Primer3 v.0.4.0) (Table S2). RT-qPCR was performed using SsoFast EvaGreen Supermix (Biorad, Hercules, CA, USA) in a CFX96 real-time PCR detection system (Biorad). The RT-qPCR thermal cycle conditions were as follows: 95 °C for 5 min, followed by 40 cycles at 95 °C for 5 s for denaturation, and 40 cycles at 60 °C for 30 s for annealing and extension. The results were expressed as Ct values normalized against GAPDH, and their gene expression levels were calculated using the 2^−ΔΔCt^ method [15].

### 2.6. Statistical Analysis

Statistical analysis was conducted using SAS 9.4 (SAS Institute, Cary, NC, USA), using gene expression data expressed as mean ± standard error (SE). The differences were analyzed using one-way ANOVA, and the statistical differences among the groups were determined using Duncan’s multiple range test. Statistical significance was considered at *p* < 0.05.

## 3. Results

**Figure 2:**
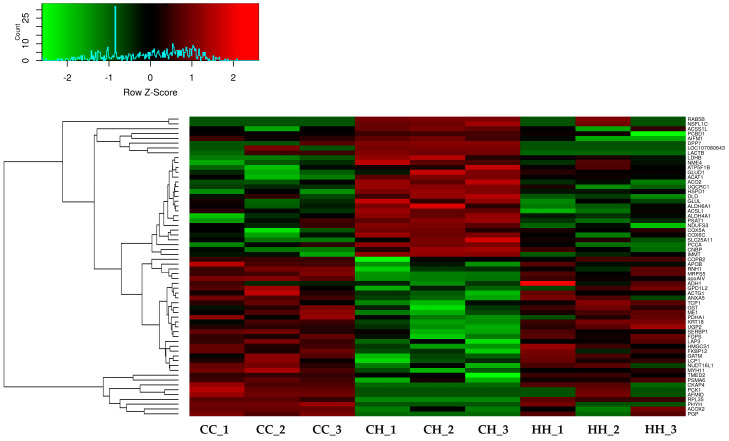
Effect of acute or early heat exposure on heatmap expression by differentially expressed proteins. CC: control group. CH: acute heat stress group. HH: early and acute heat exposure group.

A total of 2955 proteins were detected using label-free quantitation, and 991 proteins were filtered using the FDR value (FDR < 0.05). The 97 filtered proteins were significantly differentially expressed by acute HS compared with the control (Table S3). Among the 97 proteins, 62 proteins were significantly ameliorated by early heat exposure under acute HS (*p* < 0.05) (Figure 2, Table S4).

### 3.1. Bioinformatic Analysis of Differentially Expressed Proteins by Early Heat Exposure under Acute HS

**Figure 3:**
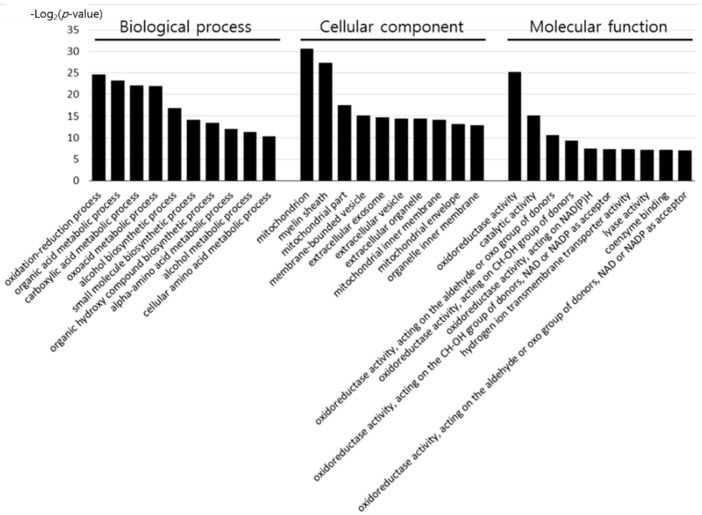
GO terms of genes encoding the differentially expressed proteins ameliorated by early heat exposure.

To determine the effect of early heat exposure on liver proteins in broilers under acute HS, the 62 DEPs ameliorated by early heat exposure were analyzed using the DAVID Bioinformatics Resources database, as shown in Figure 3 and Table S5.

Of the proteins differentially expressed by acute HS, the proteins positively affected by early heat exposure were categorized by GO terms. According to biological process, the proteins were associated with oxidation–reduction, organic acid metabolic, carboxylic acid metabolic, oxoacid metabolic, alcohol biosynthetic, small molecule biosynthetic, organic hydroxyl compound biosynthetic, alpha-amino acid metabolic, alcohol metabolic, and cellular amino acid metabolic processes. The cell components associated with the 62 proteins were the mitochondria, myelin sheath, mitochondrial part, membrane-bounded vesicle, extracellular exosome, extracellular vesicle, extracellular organelle, mitochondrial inner membrane, mitochondrial envelope, and organelle inner membrane. Furthermore, the molecular function of the proteins included oxidoreductase activity, catalytic activity, hydrogen ion transmembrane transporter activity, lyase activity, coenzyme binding, oxidoreductase activity, acting on the aldehyde or oxo group of donors, CH–OH group of donors, NAD(P)H, NAD or NADP as acceptor, oxidoreductase activity, acting on the aldehyde or oxo group of donors, and NAD or NADP as acceptor.

**Figure 4:**
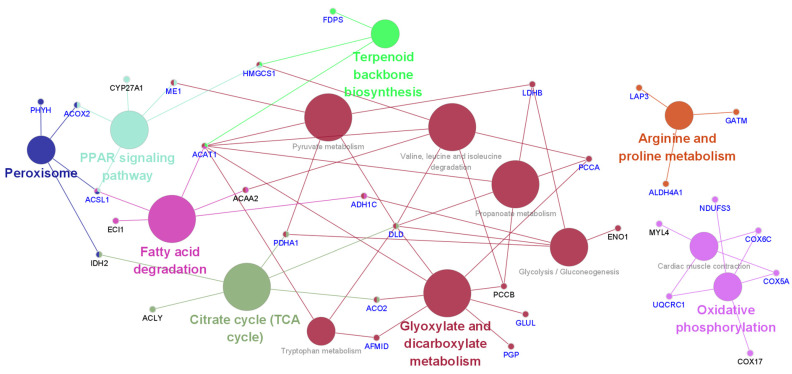
Effect of early heat exposure on the KEGG pathway of genes encoding differentially expressed proteins under acute heat stress. Blue-colored gene names: significantly altered by acute heat stress compared with the control but alleviated by early heat exposure.

The KEGG pathway analysis results of differentially expressed proteins affected by acute HS were analyzed using Cytoscape (Figure 4 and Table S6). Acute heat affected the citrate cycle (TCA cycle), terpenoid backbone biosynthesis, peroxisomes, fatty acid degradation, arginine and proline metabolism, oxidative phosphorylation, the PPAR signaling pathway, cardiac muscle contraction, glycolysis/gluconeogenesis, valine, leucine and isoleucine degradation, tryptophan metabolism, pyruvate metabolism, and propanoate metabolism. Acute HS primarily affects energy and nutrient metabolism processes. In Figure 4, the blue-colored genes indicate positive effects on genes due to early heat exposure under acute HS, demonstrating that early heat exposure may protect or reduce HS levels in broilers.

### 3.2. Validation of Differentially Expressed Proteins by Their Gene Expression

**Figure 5:**
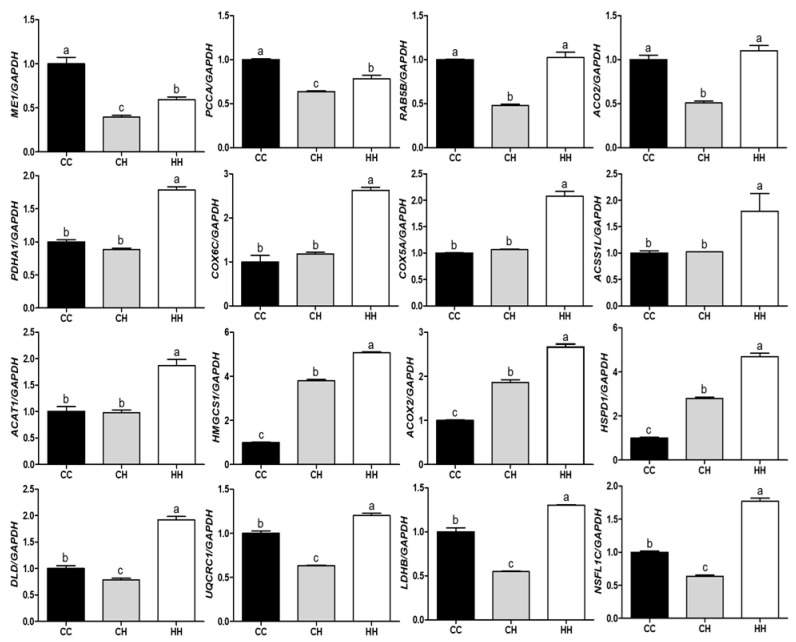
Effect of acute or early heat exposure on gene expression of differentially expressed proteins; a–c: different letters indicate significant differences (*p* < 0.05). CC: control group. CH: acute heat stress group. HH: early and acute heat exposure group. The description of each gene is presented in Table S4.

The mRNA expression of genes encoding the DEPs influenced by early heat exposure confirmed the proteomic analysis (Figure 5). Gene expression levels were normalized using *GAPDH* and the mean value of the control group. Among the 16 genes, only *ME1* showed the same pattern as the protein it encoded. Three genes, *PCCA*, *RAB5B*, and *ACO2*, were expressed in the inverse protein expression pattern. *PDHA1*, *COX6C*, *COX5A*, *ACSS1L*, and *ACAT1* genes were significantly higher in the HH group than in the CC and CH groups (*p* < 0.05), with no significant differences between the CC and CH groups. The gene expression level was also dependent on heat exposure type (acute or early), as was the case with *HMGCS1*, *ACOX2*, and *HSPD1* (*p* < 0.05). In addition, the remaining genes had significantly decreased and increased expression in the CH and HH groups, respectively, regardless of protein expression pattern (*p* < 0.05).

## 4. Discussion

HS is one of the most important environmental factors in the poultry industry. The adverse effects of HS on biological and immunological responses in animals, especially poultry, have been established [16]. Studies on HS regulation have primarily focused on reducing environmental temperature through facility management or using feed additives that can be easily applied on farms [7]. Additionally, other methods use early heat exposure to reduce HS; however, few studies have been conducted. In this study, we determined the effect of early heat exposure, hypothesizing that broilers would respond positively under late acute HS. Thus, among the various proteins that responded to acute HS, proteins that were ameliorated by early heat exposure were selected and used for analysis. Previous studies have shown that high temperatures alter animal activity and metabolic processes [17,18]. Among the responses, HS reduces the feed intake of broilers, a reduction intended to minimize heat generated during nutrient digestion and absorption. Energy intake is reduced by heat, but energy requirements increase due to demand by thermoregulatory processes to cope with HS. To compensate for this phenomenon, the energy and nutrient metabolism processes in the body are altered. In the current study, we attempted to confirm the effect of early heat exposure under acute HS at the protein level. These mechanisms were found to ameliorate protein expression by early heat exposure. Using these results, it was possible to determine the effects of acute HS and the metabolic processes mitigated by early heat exposure.

### 4.1. Carbohydrate Metabolism

HS increases respiration, which increases glucose oxidation to reduce body temperature. Acute heat exposure increases glucose and insulin levels in the body [18]. Hepatic glucose production may induce elevated fasting plasma glucose levels in response to HS [19]. Rapid depletion of liver glycogen content in response to HS is the cause of peripheral glucose increase [20]. As body temperature increases, glucose metabolism increases [18]. This is because glucose absorption increases in the intestine and kidney at high temperatures—glucose is increased by heat. In our study, HS altered six proteins (CAT1, DLD, LDHB, ME1, PCK1, and PDHA1) compared with the control, and early heat exposure ameliorated all six proteins. Among the six proteins, HS resulted in decreased PDHA1 (PDH complex) expression in pyruvate metabolism, likely leading to reduced oxidative phosphorylation of pyruvate, and pyruvate that does not enter the oxidative phosphorylation process is synthesized into l-lactate due to the increased LDHB (L-Lactate dehydrogenase B) by HS [21]. However, the expression levels of these proteins were ameliorated to a level similar to that of the control in the early heat exposure group. Based on these results, it was determined that circulating lactate was increased by acute HS, but the ability of pyruvate to circulate through the TCA cycle could be brought back to normal by early heat exposure.

### 4.2. Energy Metabolism

HS has been observed to change the direction of energy metabolism in cells [6]. An efficient ATP production process involves oxidative phosphorylation. However, these characteristics were found to increase aerobic glycolysis for ATP production and reduce ATP production through oxidative phosphorylation under HS conditions. This change is similar to the Warburg effect in cancer cells [22]. Among the DEPs associated with oxidative phosphorylation, COX5A, COX6C, NDUFS3, and UQCRC1, were increased by HS and ameliorated by early heat exposure. Oxidative phosphorylation is a synthetic ATP (energy) process consisting of an electron transport system and chemical osmotic pressure. This process is more efficient than anaerobic fermentation [23]. COX5A and COX6C are both cytochrome c oxidase families that play similar roles associated with cytochrome c oxidase activity [24]. These subunit proteins of the mitochondrial respiratory chain complex are involved in mitochondrial electron transport and transport from cytochrome c to oxygen. NDUFS3 is also critical in the electron transport system, and is involved in the assembly of electron transport system complex I [25]. When expression is impaired, mitochondrial dysfunction, reactive oxygen species production, and the apoptosis pathway of the cell are induced [26]. Proteins causally related to energy generation through the electron transport system are affected by acute HS, but are ameliorated by early heat exposure. It has been shown that acute HS attempts to satisfy the energy deficit by changing the expression of proteins vital in oxidative phosphorylation. However, in the HH group, the expression level was similar to that of the control group, and it was shown that the energy shortage phenomenon due to acute HS was not severe like in the control group.

### 4.3. Lipid Metabolism

Generally, if nutrient intake is sufficient, the utilization rate of fat in the body increases, and the oxidation of non-esterified fatty acids (NEFAs) also increases. However, despite the decrease in livestock feed intake, such as in chickens and pigs, in a high-temperature environment, lipid accumulation in carcasses increases [27,28], implying that HS or high temperatures can reduce fat breakdown and increase fat storage metabolism in animals [27]. Although the energy demand for maintaining homeostasis increases during HS, adipose tissue contribution to energy production decreases [18]. Under high temperatures, glucose may be used as the primary energy source at the expense of fat. Beta-oxidation, which metabolizes long-chain fatty acids, is a major metabolic pathway for energy homeostasis and body temperature maintenance [29]. It also plays a role in fatty acid metabolism to control the cytoplasm’s fatty acid composition in the brain. The two genes *ACO2* and *ACAT1* are involved in fatty acid metabolism, differing in their roles and positions. ACOX metabolizes long-chain fatty acids into short- or medium-chain fatty acids in peroxisomes and transfers electrons released during acyl-CoA oxidation to oxygen for H_2_O_2_ production [30]. In addition, the lack of ACOX2 expression in peroxisomes leads to increased C27-intermediate metabolite levels, resulting in oxidative stress and increased liver cell toxicity and death [31]. HS reduces feed intake, but the stress response is well explained by the increased release of fat decomposition signals (e.g., corticosterone and epinephrine). The ACAT1 protein maintains cholesterol homeostasis in cells and esterifies excess free cholesterol, converting it into cholesteryl ester (CE; a storage form of cholesterol). Increasing ACAT1 induces CE accumulation and may promote tumor proliferation [32].

### 4.4. RT- qPCR Validation

Gene expression levels were determined using RT-qPCR to confirm the proteomic assay results. The gene expression level analysis method used to verify protein expression results is a general method [33]. Therefore, we randomly selected 16 genes, but only one gene (*ME1*) was found to corroborate the protein expression level, and the remaining 15 genes differed from the protein expression level. This outcome is believed to be due to protein translation due to high temperatures [34,35].

## 5. Conclusions

In the current study, we attempted to confirm the effect of early heat exposure on acutely heat-stressed broilers at the level of proteomic changes. By analyzing the changes at the proteomic level, it was surmised that early heat exposure may controls HS. Of the 97 DEPs identified between the control and acute HS, 62 proteins were ameliorated by early heat exposure. These proteins are involved in the oxidation–reduction process, oxidative phosphorylation, fatty acids, and carbohydrate and energy metabolism. High temperatures induce rapid changes in various metabolic processes in the body to respond to or resist HS. However, the proteins maintained values similar to those of the control group due to early heat exposure, indicating that HH birds maintained homeostasis in the body by responding to HS compared with the CH group. Our results showed that early heat exposure has a positive effect on HS and can be used as primary data for further HS studies. However, verification through additional research, considering variables like the contents of energy materials (fatty acids, glucose, etc.), amount of energy (ATP), and stress hormones, needs to be conducted. Proteomic analysis with an increased experimental scale is also necessary.

## Supplementary Materials

The following are available online at https://www.mdpi.com/article/10.3390/ani11051338/s1. Table S1: Feed composition of diet; Table S2: Primer sequences for real-time qPCR; Table S3: Differentially expressed proteins by acute heat stress compared to control; Table S4: Relieved expression proteins by early heat exposure under acute heat stress; Table S5: GO terms of genes encoded alleviated proteins by early heat exposure under acute heat stress; Table S6: KEGG pathways of genes encoded differentially expressed proteins by acute heat stress.
