# Supplementary material for: Early Heat Exposure Effects on Proteomic Changes of the Broiler Liver under Acute Heat Stress
Source: Animals (Basel). 2021 May 8;11(5):1338. doi: 10.3390/ani11051338 (PMC8151403; doi:10.3390/ani11051338)
Supplement: Supplementary file 1 [file animals-11-01338-s001.zip › animals-1202650-supplementary.pdf]

Table S1. Feed composition of diet.

Item	Starter (1-20 days old)	Finisher (21-35 days old)
Crude protein (%)	20.0	19.0
Crude fat (%)	4.0	4.0
Calcium (%)	0.75	0.75
Phosphate (%)	0.70	0.70
Crude fiber (%)	6.0	5.5
Crude ash (%)	8.0	8.0
DL-methionone + cysteine + DL-methionine hydroxyl analogue (%)	0.75	0.65
Metabolizable energy (Mcal/kg)	3.00	3.05

Table S2. Primer sequences for real time qPCR.

Gene name	NCBI Reference Sequence	F/R	Sequence(5'-3')	Product size
ME1	NM_204303.1	F	GGATAGGGCTGCTTTCAACA	109
		R	GCCACGTCCCTCTGTGTATT	
ACAT1	NM_001277779.1	F	GAACAGTTACGGCTGCCAAT	113
		R	GACTATCCGTGCCAGTGGTT	
ACSS1L	XM_004941792.3	F	GGCCTTAATCTGGGAGAAGG	126
		R	ACCCTGTCACCTCGTTTCAC	
COX6C	NM_001198657.1	F	GGACTTCGAAGCCATGAGAG	87
		R	GGGATCTGTGGATCTGGAAA	
HSPD1	NM_001012916.2	F	CCAATAGTCACCGCAAACCT	131
		R	GTCACCAAAACCTGGTGCTT	
COX5A	XM_003641804.4	F	CTCCACTCCAGAGGAACTGG	106
		R	TCTGACTTGAGGCGACTGTG	
DLD	NM_001030727.2	F	GAATCCGCTTGAATCTGGAG	149
		R	TGCAGTGACTTGGTTCTTGC	
PCCA	XM_025146741.1	F	ACTCGAAGAGCAATGGGAGA	146
		R	ACGGGATGTTCAACCTGAAG	
RAB5B	NM_001199848.1	F	CGAGCGAGGAAGAGCTAAGA	75
		R	CGCTGTTATGGAGGTGAGGT	
ACO2	NM_204188.2	F	CCCTGTCCATTGCTGGTACT	100
		R	TCATCTGCATCAGGTGCTTC	
UQCRC1	NM_001005843.1	F	GCGTTACGTCCACAATGATG	135
		R	GCTGTTGCAGTCACCAGGTA	
LDHB	NM_204177.2	F	ACAGCGAGAACTGGAAGGAA	96
		R	TAAGACCAATGGCCCAGTTC	
GAPDH	NM_204305.1	F	GACGTGCAGCAGGAACACTA	112
		R	CTTGGACTTTGCCAGAGAGG	

Table S3. Differentially expressed proteins by acute heat stress compared to control.

Gi number	Protein name	MW (kDa)	Gene name	Value	
				CC	CH
46048768	alpha-enolase	49	ENO1	0.5	1.35
157954047	heat shock protein HSP 90-alpha	83	HSP90AA1	0.78	0.98
61098372	60 kDa heat shock protein, mitochondrial precursor	61	HSPD1	0.93	1.1
45383766	L-lactate dehydrogenase B chain	36	LDHB	0.8	1.21
55742654	heat shock 70 kDa protein 5	72	HSPA5	0.87	1.06
480354971	acetyl-CoA acetyltransferase, mitochondrial	44	ACAT1	0.79	1.21
60302804	long-chain-fatty-acid--CoA ligase 1	79	ACSL1	0.96	1.15
71896205	isocitrate dehydrogenase [NADP], mitochondrial	51	IDH2	0.83	1
57529492	3-ketoacyl-CoA thiolase, mitochondrial	42	ACAA2	0.8	1.05
45383738	aconitate hydratase, mitochondrial	87	ACO2	0.95	1.23
971416840	cytochrome b-c1 complex subunit 1, mitochondrial	53	UQCRC1	0.98	1.27
45382781	glutamine synthetase	42	GLUL	0.95	1.2
71897021	dihydrolipoyl dehydrogenase, mitochondrial	54	DLD	1.02	1.23
355390334	delta-1-pyrroline-5-carboxylate dehydrogenase, mitochondrial	61	ALDH4A1	0.87	1.42
478431053	enoyl-CoA delta isomerase 1, mitochondrial	40	ECI1	0.76	1.03
533112515	nucleoside diphosphate kinase, mitochondrial	21	NME4	0.9	1.21
57530041	MICOS complex subunit MIC60	79	IMMT	0.79	2.65
347543825	basigin isoform 1 precursor	42	BSG	0.91	1.07
307775405	NADPH--cytochrome P450 reductase	77	POR	0.72	1.15
478247029	protein NipSnap homolog 1	30	NIPSNAP1	0.83	1.09
363737486	cytochrome c oxidase subunit 5A, mitochondrial	16	COX5A	0.66	1.19
310772257	cytochrome c oxidase subunit 6C	9	COX6C	0.83	1.39
226437575	NADH dehydrogenase [ubiquinone] iron-sulfur protein 3, mitochondrial	29	NDUFS3	1.02	1.47
71895261	glutamine amidotransferase-like class 1 domain-containing protein 3A, mitochondrial	33	C1H21ORF33	0.88	1.13
770478845	heat shock protein beta-9	21	HSPB9	0.01	0.92
308818127	3'(2'),5'-bisphosphate nucleotidase 1	33	BPNT1	0.78	1.14
118084650	propionyl-CoA carboxylase alpha chain, mitochondrial isoform X1	79	PCCA	0.94	2.09
1390124585	propionyl-CoA carboxylase beta chain, mitochondrial isoform X1	58	PCCB	0.72	1.18
971410893	prothymosin alpha	12	PTMA	0.31	0.9
513165204	von Willebrand factor A domain-containing protein 8	212	VWA8	1	2.62
363734138	acetyl-coenzyme A synthetase 2-like, mitochondrial isoform X1	58	ACSS1L	0.63	4.83
55741590	aspartate--tRNA ligase, cytoplasmic	57	DARS	0.93	1.94
45382487	cellular nucleic acid-binding protein	15	CNBP	0.59	2.04
71895675	serine beta-lactamase-like protein LACTB, mitochondrial	58	LACTB	0.69	2.39
45382483	pterin-4-alpha-carbinolamine dehydratase	12	PCBD1	0.93	1.94
71894957	NSFL1 cofactor p47	41	NSFL1C	1	36.53
971423246	dipeptidyl peptidase 2	55	DPP7	7.12	40.22
315434208	ras-related protein Rab-5B	25	RAB5B	1	43.1
821173091	actin, cytoplasmic 2	42	ACTG1	1.1	0.85
319655768	fatty acid synthase	275	FASN	1	0.41
71896389	ATP-citrate synthase	121	ACLY	1.02	0.74
1390108559	alcohol dehydrogenase 1-like	40	LOC107053269	1.08	0.89
971437052	tubulin alpha-1B chain	49	TUBA1A1	1.3	0.76
45383538	NADP-dependent malic enzyme	64	ME1	1.09	0.72
71895873	annexin A5	36	ANXA5	1.03	0.78
363736119	glycerol-3-phosphate dehydrogenase 1-like protein	38	GPD1L2	1.08	0.88
60302740	pyruvate dehydrogenase E1 component subunit alpha, somatic form, mitochondrial precursor	45	PDHA1	1.23	0.84
476007898	dihydropyrimidinase isoform 1	58	DPYS	1.09	0.97
770478821	myosin-11	228	MYH11	1.31	0.71
874507202	UTP--glucose-1-phosphate uridylyltransferase	57	UGP2	1	0.64
56605886	plastin-2	70	LCP1	1.12	0.6

1390106173	keratin, type I cytoskeletal 18	41	KRT18	0.96	0.66
45382279	hydroxymethylglutaryl-CoA synthase, cytoplasmic	58	HMGCS1	1.12	0.53
49169816	Glutathione S-transferase	25	GST	1.01	0.65
45382065	glycine amidinotransferase, mitochondrial	48	GATM	1.17	0.7
971432899	farnesyl pyrophosphate synthase isoform X1	42	FDPS	1.09	0.63
971401941	catechol O-methyltransferase domain-containing protein 1	29	COMTD1	1.68	0.69
311213923	fibronectin precursor	272	FN1	1.45	0.86
45384392	apolipoprotein A-IV precursor	41	apoAIV	1.49	0.46
46395491	PIT54 protein precursor	55	PIT54	1.35	0.9
57530301	T-complex protein 1 subunit alpha	60	TCP1	1.09	0.65
45383077	eukaryotic initiation factor 4A-II	46	EIF4A2	1.28	0.79
71896353	heterogeneous nuclear ribonucleoprotein M	76	HNRNPM	1.22	0.9
45382029	annexin A6	75	ANXA6	2.36	0.64
113206052	apolipoprotein B precursor	523	APOB	2.37	0.6
1390102868	40S ribosomal protein S7	28	RPS7	1.48	0.71
71896825	plasminogen activator inhibitor 1 RNA-binding protein	45	SERBP1	1.14	0.76
45382147	tudor-interacting repair regulator protein precursor	34	NUDT16L1	1.11	0.69
971416599	acyl-coenzyme A oxidase 2	75	ACOX2	3.73	0.42
363736280	sterol 26-hydroxylase, mitochondrial	53	CYP27A1	1.66	0.71
1390066055	gamma-glutamylaminecyclotransferase isoform X2	19	GGACT	1.66	0.71
45382783	transgelin	27	-	1.43	0.79
57529989	ribonuclease inhibitor	50	RNH1	1.45	0.55
71897015	cytosol aminopeptidase	58	LAP3	1.29	0.62
1390063329	glycogen [starch] synthase, liver	81	GYS2	2.04	0.98
1390080620	AP-2 complex subunit beta isoform X2	106	AP2B1	1.36	0.1
309243067	39S ribosomal protein L28, mitochondrial	14	RPL28	1.34	0.77
118094989	coatomer subunit beta'	103	COPB2	1.02	0.38
45382597	cysteine and glycine-rich protein 1	20	CSRP1	1.7	0.93
472824978	phenazine biosynthesis-like domain-containing protein	32	PBLD	1.4	0.71
692314706	mitochondrial import inner membrane translocase subunit Tim8 A	11	TIMM8A	1.79	1.07
45382723	myristoylated alanine-rich C-kinase substrate	28	MARCKS	1.55	0.54
971415698	mesencephalic astrocyte-derived neurotrophic factor	20	MANF	1.81	0.62
313661410	28S ribosomal protein S5, mitochondrial	21	RPS5	1.13	0.35
45384240	serpin H1 precursor	46	SERPINH1	2.02	0.66
71894743	glycerol-3-phosphate phosphatase	33	PGP	1.64	0.03
1390119738	desmin	53	DES	2.11	0.5
82109000	FK506 bing protein 12	12	FKBP12	1.2	0.56
642945618	myosin light chain, embryonic	21	MYL4	1.79	0.61
45383588	60S ribosomal protein L35	17	RPL35	1.49	0.01
476007876	proteasome subunit alpha type-6	27	PSMA6	2.09	0.33
45384278	59 kDa 2'-5'-oligoadenylate synthase-like protein	54	OASL	40.34	1
42627796	putative ISG12(2) protein	10	ISG12(2)	1.55	0.01
971425408	kynurenine formamidase isoform X1	33	AFMID	42.39	1
57525168	transmembrane emp24 domain-containing protein 2 precursor	23	TMED2	1.02	0.34
694016461	COX17 cytochrome c oxidase copper chaperone	7	COX17	2.1	0.71
57525310	coiled-coil-helix-coiled-coil-helix domain-containing protein 2	15	CHCHD2	1.81	0.66
1390061605	phytanoyl-CoA dioxygenase, peroxisomal	39	PHYH	37.68	1

Table S4. Relieved expression proteins by early heat exposure under acute heat stress.

Gi number	Protein name	MW (kDa)	Gene name	Value		
				CC	CH	HH
971401116	Glutamate dehydrogenase 1, mitochondrial	61	GLUD1	0.89	1.08	1.01
448261627	ATP synthase subunit beta	57	ATP5F1B	0.88	1.07	1.01
61098372	60 kDa heat shock protein, mitochondrial precursor	61	HSPD1	0.93	1.1	0.99
45383766	L-lactate dehydrogenase B chain	36	LDHB	0.8	1.21	0.99
480354971	acetyl-CoA acetyltransferase, mitochondrial	44	ACAT1	0.79	1.21	0.98
60302804	long-chain-fatty-acid--CoA ligase 1	79	ACSL1	0.96	1.15	0.85
45383738	Aconitate hydratase, mitochondrial	87	ACO2	0.95	1.23	0.94
57524866	cytochrome b-c1 complex subunit 1, mitochondrial	53	UQCRC1	0.98	1.27	0.97
45382781	Glutamine synthetase	42	GLUL	0.95	1.2	0.91
71897021	Dihydrolipoyl dehydrogenase	54	DLD	1.02	1.23	0.9
355390334	delta-1-pyrroline-5-carboxylate dehydrogenase, mitochondrial	61	ALDH4A1	0.87	1.42	0.95
513188218	methylmalonate-semialdehyde dehydrogenase [acylating], mitochondrial	55	ALDH6A1	0.98	1.18	0.93
533112515	Nucleoside diphosphate kinase	21	NME4	0.9	1.21	1.04
363744581	Phosphoserine aminotransferase	41	PSAT1	0.99	1.46	0.88
57530041	MICOS complex subunit MIC60	79	IMMT	0.79	2.65	0.87
363737486	Cytochrome c oxidase subunit 5A	16	COX5A	0.66	1.19	0.98
310772257	cytochrome c oxidase subunit 6C	9	COX6C	0.83	1.39	0.91
226437575	Complex1_30kDa domain-containing protein	29	NDUFS3	1.02	1.47	0.67
974005349	C-factor-like	28	LOC107080643	6.93	19.22	1
1.39E+09	Solute carrier family 25 member 11	25	SLC25A11	0.79	1.43	0.99
118084650	propionyl-CoA carboxylase alpha chain, mitochondrial isoform X1	79	PCCA	0.94	2.09	0.68
55926141	apoptosis-inducing factor 1, mitochondrial	75	AIFM1	1	2.17	0.14
363734138	acetyl-coenzyme A synthetase 2-like, mitochondrial isoform X1	58	ACSS1L	0.63	4.83	1.04
45382487	Cellular nucleic acid-binding protein	15	CNBP	0.59	2.04	0.98
71895675	serine beta-lactamase-like protein LACTB, mitochondrial	58	LACTB	0.69	2.39	0.05
45382483	Pterin-4-alpha-carbinolamine dehydratase	12	PCBD1	0.93	1.94	0.68
71894957	NSFL1 cofactor p47	41	NSFL1C	1	36.53	10.35
971423246	dipeptidyl peptidase 2	55	DPP7	7.12	40.22	1
315434208	Ras-related protein Rab-5B	25	RAB5B	1	43.1	16.13
821173091	Actin, cytoplasmic 2	42	ACTG1	1.1	0.85	1.04
762006017	Alcohol dehydrogenase 1	40	ADH1	1.08	0.89	1.02
45383538	Malic enzyme	64	ME1	1.09	0.72	1.07
71895873	Annexin A5	36	ANXA5	1.03	0.78	0.97
363736119	Glycerol-3-phosphate dehydrogenase [NAD(+)]	38	GPD1L2	1.08	0.88	1.03
60302740	Pyruvate dehydrogenase E1 component subunit alpha	45	PDHA1	1.23	0.84	1.07
770478821	Myosin-11	228	MYH11	1.31	0.71	1.01
874507202	UTP--glucose-1-phosphate uridylyltransferase	57	UGP2	1	0.64	1.15
56605886	Uncharacterized protein	70	LCP1	1.12	0.6	1.11
1.39E+09	Keratin 18	41	KRT18	0.96	0.66	1.08
45382279	hydroxymethylglutaryl-CoA synthase, cytoplasmic	58	HMGCS1	1.12	0.53	1.16
49169816	Glutathione S-transferase	25	GST	1.01	0.65	1.09
45382065	Glycine amidinotransferase, mitochondrial	48	GATM	1.17	0.7	1.15
971432899	Farnesyl pyrophosphate synthase	42	FDPS	1.09	0.63	1.12
3645997	Apolipoprotein AIV	41	apoAIV	1.49	0.46	1.07
57530301	T-complex 1	60	TCP1	1.09	0.65	1.2
102221132	Apolipoprotein B	523	APOB	2.37	0.6	1.3
71896825	plasminogen activator inhibitor 1 RNA-binding protein	45	SERBP1	1.14	0.76	1.04
45382147	Tudor-interacting repair regulator protein	34	NUDT16L1	1.11	0.69	0.98

971416599	acyl-coenzyme A oxidase 2	75	ACOX2	3.73	0.42	1.76
57529989	Ribonuclease/angiogenin inhibitor 1	50	RNH1	1.45	0.55	1.03
71897015	cytosol aminopeptidase	58	LAP3	1.29	0.62	1.1
118094989	Coatomer subunit beta'	103	COPB2	1.02	0.38	0.94
313661410	28S ribosomal protein S5, mitochondrial	21	MRPS5	1.13	0.35	0.99
71894743	Glycerol-3-phosphate phosphatase	33	PGP	1.64	0.03	0.73
14211655	FK506 bing protein 12	12	FKBP12	1.2	0.56	1.08
971372403	Cytoskeleton associated protein 4	57	CKAP4	1.65	0.05	0.75
45383588	60S ribosomal protein L35	17	RPL35	1.49	0.01	1.31
476007876	proteasome subunit alpha type-6	27	PSMA6	2.09	0.33	1.32
45382671	Phosphoenolpyruvate carboxykinase, cytosolic [GTP]	70	PCK1	25.49	1	6.41
971425408	Kynurenine formamidase	33	AFMID	42.39	1	12.57
57525168	Transmembrane p24 trafficking protein 2	23	TMED2	1.02	0.34	0.93
1390061605	Phytanoyl-CoA 2-hydroxylase	39	PHYH	37.68	1	10.77

Table S5. GO terms of genes encoded alleviated proteins by early heat exposure under acute heat stress.

GO Term	%	P-value	Genes
		Biological process	
oxidation-reduction process	22	4E-08	ACAT1, ACO2, ACOX2, ADH1, APOA4, COX5A, DLD, GLUD1, LDHB, ME1, PCK1, PCBD1, UQCRFS1
organic acid metabolic process	25.4	1E-07	ACAT1, ACO2, ACOX2, LOC423347, ALDH4A1, ALDH6A1, APOA4, AFMID, GLUD1, GLUL, LDHB, ME1, PCK1, PSAT1, PDHA1
carboxylic acid metabolic process	23.7	2.3E-07	ACAT1, ACO2, ACOX2, LOC423347, ALDH4A1, ALDH6A1, APOA4, GLUD1, GLUL, LDHB, ME1, PCK1, PSAT1, PDHA1
oxoacid metabolic process	23.7	2.5E-07	ACAT1, ACO2, ACOX2, LOC423347, ALDH4A1, ALDH6A1, APOA4, GLUD1, GLUL, LDHB, ME1, PCK1, PSAT1, PDHA1
alcohol biosynthetic process	10.2	9.1E-06	HMGCS1, APOA3, APOB, FDPS, PCK1, PGP
small molecule biosynthetic process	15.3	0.00006	HMGCS1, ALDH4A1, APOA4, APOB, FDPS, GLUL, PCK1, PGP, PSAT1
organic hydroxy compound biosynthetic process	10.2	0.0001	HMGCS1, APOA4, APOB, FDPS, PCK1, PGP
alpha-amino acid metabolic process	10.2	0.00025	ALDH4A1, AFMID, GLUD1, GLUL, PCK1, PSAT1
alcohol metabolic process	10.2	0.00043	HMGCS1, APOA4, APOB, FDPS, PCK1, PGP
cellular amino acid metabolic process	10.2	0.00085	ALDH4A1, ALDH6A1, GLUD1, GLUL, PCK1, PSAT1
		Cellular component	
mitochondrion	42.4	6E-10	ATP5B, NDUFS3, NME4, ACAT1, ACO2, ACOX2, ACSL1, ALDH4A1, ALDH6A1, AIFM1, COX5A, COX6C, DLD, GLUD1, GLUL, GATM, HSPD1, IMMT, LACTB, LDHB, LAP3, MRPS5, PCCA, PDHA1, UQCRFS1
myelin sheath	18.6	6.2E-09	ATP5B, NDUFS3, ACO2, LOC776816, COX5A, DLD, HSPD1, IMMT, LDHB, TCP1, UQCRFS1
mitochondrial part	22	5.3E-06	ATP5B, NDUFS3, ACAT1, ACO2, AIFM1, COX5A, COX6C, GLUD1, GATM, HSPD1, IMMT, MRPS5, UQCRFS1
membrane-bounded vesicle	44.1	0.00003	ATP5B, FKBP1A, RAB5B, UGP2, ACAT1, LOC776816, ALDH6A1, ANXA5, APOA4, APOB, COPB2, COX5A, DLD, DPP7, GATM, HSPD1, LDHB, LAP3, LCP1, MYH11, PCK1, PSAT1, PSMA6, PCBD1, RNH1, TCP1
extracellular exosome	39	0.00004	ATP5B, FKBP1A, UGP2, ACAT1, LOC776816, ALDH6A1, ANXA5, APOA4, APOB, COX5A, DPP7, GATM, HSPD1, LDHB, LAP3, LCP1, MYH11, PCK1, PSAT1, PSMA6, PCBD1, RNH1, TCP1
extracellular vesicle	39	0.00005	ATP5B, FKBP1A, UGP2, ACAT1, LOC776816, ALDH6A1, ANXA5, APOA4, APOB, COX5A, DPP7, GATM, HSPD1, LDHB, LAP3, LCP1, MYH11, PCK1, PSAT1, PSMA6, PCBD1, RNH1, TCP1
extracellular organelle	39	0.00005	ATP5B, FKBP1A, UGP2, ACAT1, LOC776816, ALDH6A1, ANXA5, APOA4, APOB, COX5A, DPP7, GATM, HSPD1, LDHB, LAP3, LCP1, MYH11, PCK1, PSAT1, PSMA6, PCBD1, RNH1, TCP1
mitochondrial inner membrane	15.3	0.00006	ATP5B, NDUFS3, ACAT1, COX5A, COX6C, GATM, HSPD1, IMMT, UQCRFS1
mitochondrial envelope	16.9	0.00012	ATP5B, NDUFS3, ACAT1, AIFM1, COX5A, COX6C, GATM, HSPD1, IMMT, UQCRFS1
organelle inner membrane	15.3	0.00014	ATP5B, NDUFS3, ACAT1, COX5A, COX6C, GATM, HSPD1, IMMT, UQCRFS1
		Molecular Function	
oxidoreductase activity	28.8	2.6E-08	NDUFS3, ACOX2, ADH1, ALDH4A1, ALDH6A1, AIFM1, COX5A, COX6C, DLD, GLUD1, GPD1L2, LDHB, ME1, PCK1, PCBD1, PDHA1, UQCRFS1
catalytic activity	66.1	0.00003	HMGCS1, ATP5B, FKBP1A, NDUFS3, NME4, UGP2, ACAT1, ACO2, ACOX2, ACSL1, LOC423347, ADH1, ALDH4A1, ALDH6A1, AIFM1, AFMID, COX5A, COX6C, DLD, DPP7, FDPS,

			GLUD1, GLUL, GSTA3, GPD1L2, GATM, LDHB, LAP3, ME1, MYH11, NUDT16L1, PCK1, PGP, PSAT1, PCCA, PSMA6, PCBD1, PDHA1, UQCRFS1
oxidoreductase activity, acting on the aldehyde or oxo group of donors	6.8	0.0007	ALDH4A1, ALDH6A1, DLD, PDHA1
oxidoreductase activity, acting on CH-OH group of donors	8.5	0.0016	ADH1, GPD1L2, LDHB, ME1, PCK1
oxidoreductase activity, acting on NAD(P)H	6.8	0.0061	NDUFS3, AIFM1, DLD, PCK1
oxidoreductase activity, acting on the CH-OH group of donors, NAD or NADP as acceptor	6.8	0.007	ADH1, GPD1L2, LDHB, ME1
hydrogen ion transmembrane transporter activity	6.8	0.007	ATP5B, COX5A, COX6C, UQCRFS1
lyase activity	8.5	0.0073	ACO2, GLUL, ME1, PCK1, PCBD1
coenzyme binding	8.5	0.0073	ACOX2, AIFM1, DLD, GPD1L2, ME1
oxidoreductase activity, acting on the aldehyde or oxo group of donors, NAD or NADP as acceptor	5.1	0.0084	ALDH4A1, ALDH6A1, DLD

Table S6. KEGG pathways of genes encoded differentially expressed proteins by acute heat stress.

Term	p-value	% associated genes	genes
Citrate cycle (TCA cycle)	< 0.001	17.86	*ACLY*, ***ACO2***, ***DLD***, *IDH2*, ***PDHA1***
Terpenoid backbone biosynthesis	0.001	15.79	***ACAT1***, ***FDPS***, ***HMGCS1***
PPAR signaling pathway	< 0.001	7.94	***ACOX2***, ***ACSL1***, *CYP27A1*, ***HMGCS1***, ***ME1***
Peroxisome	0.01	5.06	***ACOX2***, ***ACSL1***, *IDH2*, ***PHYH***
Fatty acid degradation	< 0.001	14.71	*ACAA2*, ***ACAT1***, ***ACSL1***, ***ADH1C***, *ECI1*
Arginine and proline metabolism	0.011	7.14	***ALDH4A1***, ***GATM***, ***LAP3***
Oxidative phosphorylation	0.008	4.31	*COX17*, ***COX5A***, ***COX6C***, ***NDUFS3***, ***UQCRC1***
Cardiac muscle contraction	0.006	5.71	***COX5A***, ***COX6C***, *MYL4*, ***UQCRC1***
Glycolysis / Gluconeogenesis	< 0.001	9.62	***ADH1C***, ***DLD***, *ENO1*, ***LDHB***, ***PDHA1***
Valine, leucine and isoleucine degradation	< 0.001	13.64	*ACAA2*, ***ACAT1***, ***DLD***, ***HMGCS1***, ***PCCA***, *PCCB*
Tryptophan metabolism	0.007	8.11	***ACAT1***, ***AFMID***, ***DLD***
Pyruvate metabolism	< 0.001	15.63	***ACAT1***, ***DLD***, ***LDHB***, ***ME1***, ***PDHA1***
Glyoxylate and dicarboxylate metabolism	< 0.001	25.81	***ACAT1***, ***ACO2***, ***AFMID***, ***DLD***, ***GLUL***, ***PCCA***, *PCCB*, ***PGP***
Propanoate metabolism	< 0.001	15.63	***ACAT1***, ***DLD***, ***LDHB***, ***PCCA***, *PCCB*

Bold type genes showed that significantly changed by acute heat stress compared to control but alleviated by early heat exposure.
